# Diagnostic value of serum inflammatory markers in predicting early refractoriness of transarterial chemoembolization in patients with Barcelona Clinic Liver Cancer Stage 0, A, and B hepatocellular carcinoma

**DOI:** 10.1590/1414-431X2024e13661

**Published:** 2024-09-06

**Authors:** Junhui Yang, Yunjie Zhang, Yifan Kong, Jiawei Lin, Guoqing Zhu, Hao Zhang, Zhijie Yu, Pixu Liu, Jinglin Xia

**Affiliations:** 1 Zhejiang Key Laboratory of Intelligent Cancer Biomarker Discovery and Translation, First Affiliated Hospital of Wenzhou Medical University, Wenzhou, Zhejiang Province, China; 2 Department of Interventional Radiology, The First Affiliated Hospital of Wenzhou Medical University, Wenzhou, Zhejiang Province, China; 3 Department of Gastroenterology, The Wenzhou Central Hospital, Wenzhou, Zhejiang Province, China; 4 Liver Cancer Institute, Zhongshan Hospital of Fudan University, Shanghai, China

**Keywords:** Hepatocellular carcinoma, Transarterial chemoembolization, TACE refractoriness, Inflammatory markers, Predictive model

## Abstract

Transarterial chemoembolization (TACE) is an established therapeutic strategy for intermediate stage Barcelona Clinic Liver Cancer (BCLC) hepatocellular carcinoma (HCC). However, patients who are early refractory to TACE may not benefit from repeated TACE treatment. Our primary objective was to assess the diagnostic value of inflammatory markers in identifying early TACE refractory for patients with early (BCLC 0 and A) or intermediate (BCLC B) stage HCC. We retrospectively reviewed the HCC patients who underwent TACE as the initial treatment in two hospitals. Patients with early TACE refractoriness had significantly poorer median overall survival (OS) (16 *vs* 40 months, P<0.001) and progression-free survival (PFS) (7 *vs* 23 months, P<0.001) compared to TACE non-refractory patients. In the multivariate regression analysis, tumor size (P<0.001), bilobular invasion (P=0.007), high aspartate aminotransferase-to-platelet ratio index (APRI) (P=0.007), and high alpha fetoprotein (AFP) level (P=0.035) were independent risk factors for early TACE refractoriness. The predictive model showcasing these factors exhibited high ability proficiency, with an area under curve (AUC) of 0.833 (95%CI=0.774-0.892) in the training cohort, 0.750 (95%CI: 0.640-0.861) in the internal-validation cohort, and 0.733 (95%CI: 0.594-0.872) in the external-validation cohort. Calibration curve analysis revealed good agreement between the actual and predicted probabilities of early TACE refractoriness. Our preliminary study estimated the potential value of inflammatory markers in predicting early TACE refractoriness and provides a predictive model to assist in identifying patients who may not benefit from repeat TACE treatment.

## Introduction

Hepatocellular carcinoma (HCC), a tumor characterized by high heterogeneity, is the third most common cause of cancer-related deaths globally (1). At diagnosis, over 80% of HCC patients are at an intermediate or advanced stage, missing the opportunity of curative treatments, and often exhibit resistance to conventional chemotherapy and radiotherapy (2). A previous global study involving 14 countries and 18,031 patients demonstrated that transarterial embolization (TACE) is the most widely used treatment in all stages of Barcelona Clinic Liver Cancer (BCLC) (3). As a locoregional interventional therapy, TACE uses the combination of arterial injection of anticancer drugs with a vector (lipiodol or embolization microspheres) and arterial embolization by gelatin or microspheres. Repeat TACE procedures are employed to maximize tumor response and improve outcome (4). However, not all patients experience advantages from undergoing additional TACE procedures because of the occurrence of drug-induced liver toxicity and tumor heterogeneity. To address the issue of ineffective repeated TACE treatments, the concept of TACE failure/refractoriness was firstly introduced by the Japan Society of Hepatology (JSH). According to the JSH standard, TACE refractoriness is defined as: 1) the occurrence of two or more consecutive insufficient responses (live lesion >50%) or appearance of new lesions in the treatment zone, even following alterations in chemotherapeutic drugs or reassessment of the feeding arteries; 2) consistently elevated tumor markers (even with a temporary decrease); 3) appearance of vascular invasion; or 4) appearance of extrahepatic metastasis (5,6). A real-world study demonstrated that the median overall survival (OS) of the TACE refractory patients was significantly poorer compared with non-refractory patients (21 *vs* 34 months, P=0.002) (7).

Inflammation plays an important role in cancer, which has recently become a research hotspot. Serum inflammatory markers including neutrophil-to-lymphocyte ratio (NLR), gamma-glutamyl transpeptidase-to-lymphocyte ratio (GLR), gamma-glutamyl transpeptidase-to-platelet ratio (GPR), lymphocyte-to-monocyte ratio (LMR), aspartate aminotransferase-to-platelet ratio index (APRI), and aspartate aminotransferase-to-neutrophil ratio index (ANRI) can reflect systemic inflammatory status (8). Compared with tumor imaging characteristics and other serum markers, inflammatory markers composed of inflammation-related hematologic and biochemical indicators are cheaper and easier to obtain. Recent studies have shown that inflammatory markers have vital value for the therapeutic effect and prognosis prediction of HCC (9-11). However, the relationship between inflammatory markers and early TACE refractoriness remains to be thoroughly explored.

Our study aimed to investigate the value of serum inflammatory markers in determining the TACE refractory status and subsequently develop and validate an early TACE refractoriness diagnostic model for patients with early/intermediate stage HCC.

## Material and Methods

### Patients

This study was approved by the Ethics Committee of the First Affiliated Hospital of Wenzhou Medical University (approval number: KY2023-R132) and followed the Declaration of Helsinki (as revised in 2013). As the present study was retrospective, the need for patient consent was exempted.

**Figure 1:**
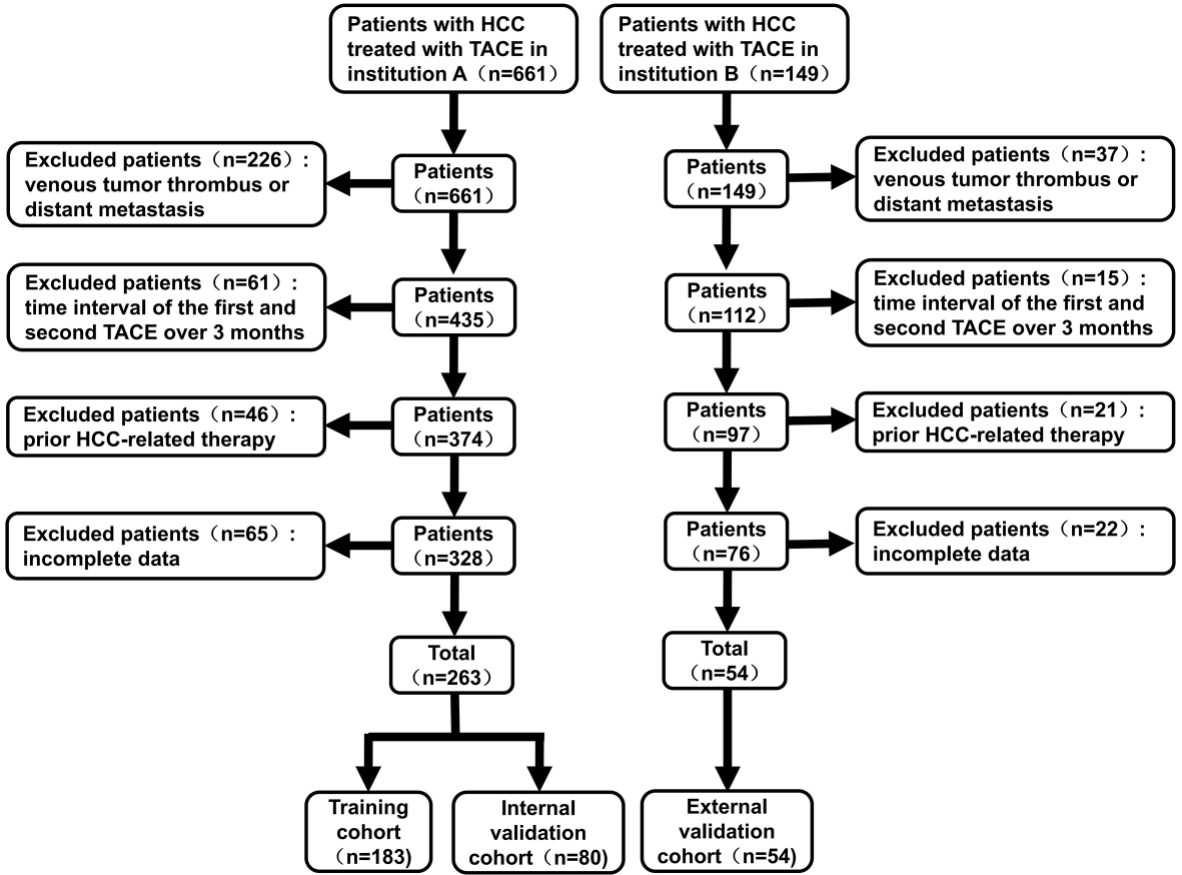
Flowchart for patient screening. HCC: hepatocellular carcinoma; TACE: transarterial chemoembolization.

We analyzed 661 unresectable HCC patients, who initially underwent TACE at Hospital A from January, 2017 to December, 2021 (Figure 1). The inclusion criteria were as follows: 1) TACE as the initial treatment with at least 2 sessions of TACE (patients achieving complete response after initial treatment were also included); 2) Child Pugh A or B level; and 3) Eastern Cooperative Oncology Group (ECOG) performance status 0/1. On the other hand, the exclusion criteria were: 1) venous tumor thrombus or distant metastasis (n=226); 2) a time interval of TACE treatments over 3 months (n=61); 3) prior HCC-related therapy containing radical resection, systematic therapy, and other locoregional therapies, (n=46); and 4) incomplete data (n=65). Finally, 263 patients were randomly assigned to the training cohort (n=183, 70%) or the internal verification cohort (n=80, 30%) with R package “caret” procedure. Additionally, 149 HCC patients who underwent TACE as an initial therapy between August 2019 and December 2022 from hospital B were collected for external validation. After applying the same screening criteria, 54 patients were selected for the external cohort.

### Data collection

The clinical data were extracted within 2 weeks prior to the first TACE to estimate the independent predictor for TACE refractory patients. The demographic and laboratory data contained age at diagnosis, gender, BCLC stage, Child Pugh score, hepatitis B history, levels of alpha fetoprotein (AFP), NLR, GLR, GPR, LMR, APRI, ANRI, aspartate aminotransferase (AST), alanine aminotransferase (ALT), γ-glutamyl transpeptidase (GGT), monocyte count, lymphocyte count, neutrophil count, and platelet count. The radiological data contained tumor size, tumor number, bilobular invasion, ascites, and liver cirrhosis.

### Treatment procedure

Before administration, clinicians discussed and chose the appropriate TACE procedures for each patient. A modified Seldinger technique was adopted for right femoral artery evaluation, and a microcatheter enabled selective examination of the tumor-feeding artery. Conventional TACE was a mix of 10 to 50 mg of doxorubicin or other chemotherapeutic drug, combined with 2 to 20 mL iodized oil as an emulsifying agent. The dosage of drugs and iodized oil was usually determined in combination with the tumor load, blood supply artery of the tumor, and the patient's liver function. Gelfoam sponge slurries were employed for the embolization of the tumor feeders. DEB-TACE involved a doxorubicin dosage varying from 30 to 70 mg and drug-eluting beads (DEB) of a diameter ranging from 100 to 300 μm and 300 to 500 μm. After fully mixed and diluted with drugs, the drug-eluting beads were injected to embolize the tumor feeders without additional embolization and continuously release antineoplastic drugs to kill tumors.

### Follow-up and definition

Early TACE refractoriness was the primary outcome of the study, and the secondary outcomes were OS and PFS. To estimate the treatment response of TACE, dynamic enhanced CT or MR images were obtained within 1-3 months after the initial two TACE treatments. Besides, regular imaging examinations were performed to continuously evaluate tumor lesions after two courses of treatment.

The diagnosis of TACE refractoriness was determined according to the JSH standard (5,6). In addition, patients exhibiting TACE refractoriness following the first two continuous TACE sessions were defined as early TACE refractoriness.

### Statistical analysis

Data analysis was done using R software (v4.2.2; R Core Team), and data are reported as mean and standard deviation. Categorical and continuous variables were compared with the Fisher's test and the Student's *t*-test, respectively. Univariate and multivariate analyses were conducted using logistic regression models. An R package called “rms” was employed to construct a nomogram incorporating the independent risk factors associated with early TACE refractoriness. The model's predictive performance was tested by plotting receiver operating characteristic (ROC) curves. The diagnostic model's consistency was estimated by calibration curves, and the goodness of fit by Hosmer-Lemeshow test. Kaplan-Meier analysis was performed for survival analysis and compared using the log-rank test. A significance level of 0.05 was used to determine statistical significance.

## Results

### Clinical characteristics of patients

In total, 263 patients in hospital A were ultimately selected and assigned to the training cohort (n=183) and internal validation cohort (n=80). Baseline characteristics between the two cohorts exhibited no substantial differences (Table 1). Among the patients from hospital A, 19% (n=50) had a high tumor load (tumor size >100mm), 45.9% (n=137) had more than one lesion, 27.4% (n=72) had bilobular invasion, and 23.2% (n=61) exhibited an elevated AFP level (>400 ng/mL). According to BCLC staging system, 10.6% patients (n=28) were at stage 0, 46.0% patients (n=121) at stage A, and 43.3% patients (n=114) at stage B. According to Child Pugh score, 79.8% patients (n=210) were in class A and 20.2% patients (n=53) were in class B. Additionally, 54 patients in hospital B were included as the external validation cohort. Several disparities were observed in baseline characteristics between the two cohorts, including liver cirrhosis (P=0.020), hepatitis B infection (P=0.017), and Child Pugh score (P=0.014), as presented in Table 2. The external verification cohort was established to verify the accuracy of the model without any interference in subsequent analyses.

**Table 1:** Comparison of baseline characteristics between the training and internal validation cohorts.

Characteristics	Overall(n=263)	Training cohort(n=183)	Internal validation cohort (n=80)	P value
Age	62.7±12.0	62.5±12.6	63.3±10.5	0.614
Gender (%)				1.000
Male	221 (84.0)	154 (84.2)	67 (83.8)	
Female	42 (16.0)	29 (15.8)	13 (16.2)	
Tumor size (%)				0.067
≤50 mm	126 (47.9)	79 (43.2)	47 (58.8)	
50-100 mm	87 (33.1)	66 (36.1)	21 (26.3)	
>100 mm	50 (19.0)	38 (20.8)	12 (15.0)	
Tumor number (%)				0.080
Solitary	116 (44.1)	84 (45.9)	32 (40.0)	
2-3	90 (34.2)	55 (30.1)	35 (43.8)	
>3	57 (21.7)	44 (24.0)	13 (16.3)	
Bilobular invasion (%)				0.279
No	191 (72.6)	137 (74.9)	54 (67.5)	
Yes	72 (27.4)	46 (25.1)	26 (32.5)	
BCLC stage (%)				0.392
0	28 (10.6)	22 (12.0)	6 (7.5)	
A	121 (46.0)	80 (43.7)	41 (51.3)	
B	114 (43.3)	81 (44.3)	33 (41.3)	
Ascites (%)				0.821
Absence	198 (75.3)	139 (76.0)	59 (73.8)	
Presence	65 (24.7)	44 (24.0)	21 (26.3)	
Liver cirrhosis (%)				0.195
Absence	89 (33.8)	67 (36.6)	22 (27.5)	
Presence	174 (66.2)	116 (63.4)	58 (72.5)	
AFP (%)				0.764
≤400 ng/mL	202 (76.8)	142 (77.6)	60 (75.0)	
>400 ng/mL	61 (23.2)	61 (22.4)	20 (25.0)	
Child Pugh score (%)				0.899
A	210 (79.8)	147 (80.3)	63 (78.8)	
B	53 (20.2)	36 (19.7)	17 (21.3)	
Hepatitis B (%)				0.152
Absence	131 (49.8)	97 (53.0)	34 (42.5)	
Presence	132 (50.2)	86 (47.0)	46 (57.5)	
Monocyte (10ˆ9/L)	0.557±0.569	0.573±0.517	0.522±0.674	0.543
Lymphocyte (10ˆ9/L)	1.41±0.633	1.41±0.572	1.42±0.759	0.921
Neutrophil (10ˆ9/L)	3.64±1.90	3.76±2.12	3.36±1.24	0.052
Platelet (10ˆ9/L)	170±94.7	176±94.6	156±93.9	0.124
ALT (U/L)	41.1±37.4	41.6±38.0	39.9±36.2	0.722
AST (U/L)	52.1±41.1	52.4±43.0	51.3±36.6	0.834
GGT (U/L)	181±416	164±211	220±685	0.472
NLR	3.19±4.46	3.30±5.20	2.95±1.93	0.433
GLR	158±431	135±174	210±737	0.374
GPR	1.22±2.16	1.05±1.16	1.61±3.49	0.171
LMR	3.16±1.71	3.05±1.70	3.40±1.72	0.136
APRI	0.48±1.18	0.39±0.36	0.69±2.07	0.202
ANRI	17.7±15.8	17.3±15.6	18.6±16.3	0.553
TACE refractoriness (%)				1.000
Absence	169 (64.3)	118 (64.5)	51 (63.8)	
Presence	94 (35.7)	65 (35.5)	29 (36.3)	

Data are reported as mean and SD or number and percent. Fisher’s test or *t*-test. BCLC: Barcelona Clinic Liver Cancer; AFP: alpha fetoprotein; ALT: alanine aminotransferase; AST: aspartate aminotransferase; ANRI: aspartate aminotransferase-to-neutrophil ratio index; APRI: aspartate aminotransferase-to-platelet ratio index; NLR: neutrophil-to-lymphocyte ratio; LMR: lymphocyte-to-monocyte ratio: GGT: γ-glutamyl transpeptidase, GPR: gamma-glutamyl transpeptidase-to-platelet ratio; GLR: gamma-glutamyl transpeptidase-to-lymphocyte ratio; TACE: transarterial chemoembolization.

**Table 2:** Comparison of baseline characteristics between the training and external validation cohorts.

Characteristics	Overall(n=237)	Training cohort(n=183)	External validation cohort (n=54)	P value
Age	63.2±12.2	62.5±12.6	65.5±10.9	0.087
Gender (%)				0.235
Male	195 (82.3)	154 (84.2)	41 (75.9)	
Female	42 (17.7)	29 (15.8)	13 (24.1)	
Tumor size (%)				0.226
≤50 mm	109 (46.0)	79 (43.2)	30 (55.6)	
50-100 mm	83 (35.0)	66 (36.1)	17 (31.5)	
>100 mm	45 (19.0)	38 (20.8)	7 (13.0)	
Tumor number (%)				0.156
Solitary	107 (45.1)	84 (45.9)	23 (42.6)	
2-3	78 (32.9)	55 (30.1)	23 (42.6)	
>3	52 (21.9)	44 (24.0)	8 (14.8)	
Bilobular invasion (%)				0.590
No	180 (75.9)	137 (74.9)	43 (79.6)	
Yes	57 (24.1)	46 (25.1)	11 (20.4)	
BCLC stage (%)				0.827
0	30 (12.7)	22 (12.0)	8 (14.8)	
A	104 (43.9)	80 (43.7)	24 (44.4)	
B	103 (43.5)	81 (44.3)	22 (40.7)	
Ascites (%)				0.146
Absence	174 (73.4)	139 (76.0)	35 (64.8)	
Presence	63 (26.6)	44 (24.0)	19 (35.2)	
Liver cirrhosis (%)				0.020
Absence	77 (32.5)	67 (36.6)	10 (18.5)	
Presence	160 (67.5)	116 (63.4)	44 (81.5)	
AFP (%)				0.673
≤400 ng/ml	186 (78.5)	142 (77.6)	44 (81.5)	
>400 ng/ml	51 (21.5)	61 (22.4)	10 (18.5)	
Child Pugh score (%)				0.014
A	181 (76.4)	147 (80.3)	34 (63.0)	
B	56 (23.6)	36 (19.7)	20 (37.0)	
Hepatitis B (%)				0.017
Absence	115 (48.5)	97 (53.0)	18 (33.3)	
Presence	122 (51.5)	86 (47.0)	36 (66.7)	
Monocyte (10ˆ9/L)	0.561±0.473	0.573±0.517	0.520±0.276	0.324
Lymphocyte (10ˆ9/L)	1.38±0.594	1.41±0.572	1.28±0.659	0.211
Neutrophil (10ˆ9/L)	3.76±2.36	3.76±2.12	3.74±3.06	0.963
Platelet (10ˆ9/L)	170±96.4	176±94.6	148±100	0.077
ALT (U/L)	40.9±40.1	41.6±38.0	38.4±46.6	0.637
AST (U/L)	52.3±48.3	52.4±43.0	52.0±63.7	0.962
GGT (U/L)	155±193	164±211	126±107	0.082
NLR	3.36±4.81	3.30±5.20	3.59±3.17	0.607
GLR	132±166	135±174	121±133	0.527
GPR	1.05±1.10	1.05±1.16	1.05±0.93	0.983
LMR	2.98±1.64	3.05±1.70	2.74±1.39	0.168
APRI	0.46±1.06	0.39±0.36	0.68±2.09	0.330
ANRI	17.9±19.5	17.3±15.6	18.6±27.6	0.820
TACE refractoriness (%)				0.966
Absence	152 (64.1)	118 (64.5)	34 (63.0)	
Presence	85 (35.9)	65 (35.5)	20 (37.0)	

Data are reported as mean and SD or number and percent. Fisher’s test or *t*-test. BCLC: Barcelona Clinic Liver Cancer; AFP: alpha fetoprotein; ALT: alanine aminotransferase; AST: aspartate aminotransferase; ANRI: aspartate aminotransferase-to-neutrophil ratio index; APRI: aspartate aminotransferase-to-platelet ratio index; NLR: neutrophil-to-lymphocyte ratio; LMR: lymphocyte-to-monocyte ratio: GGT: γ-glutamyl transpeptidase; GPR: gamma-glutamyl transpeptidase-to-platelet ratio; GLR: gamma-glutamyl transpeptidase-to-lymphocyte ratio; TACE: transarterial chemoembolization.

### Patterns and prognosis of patients with early TACE refractoriness

**Figure 2:**
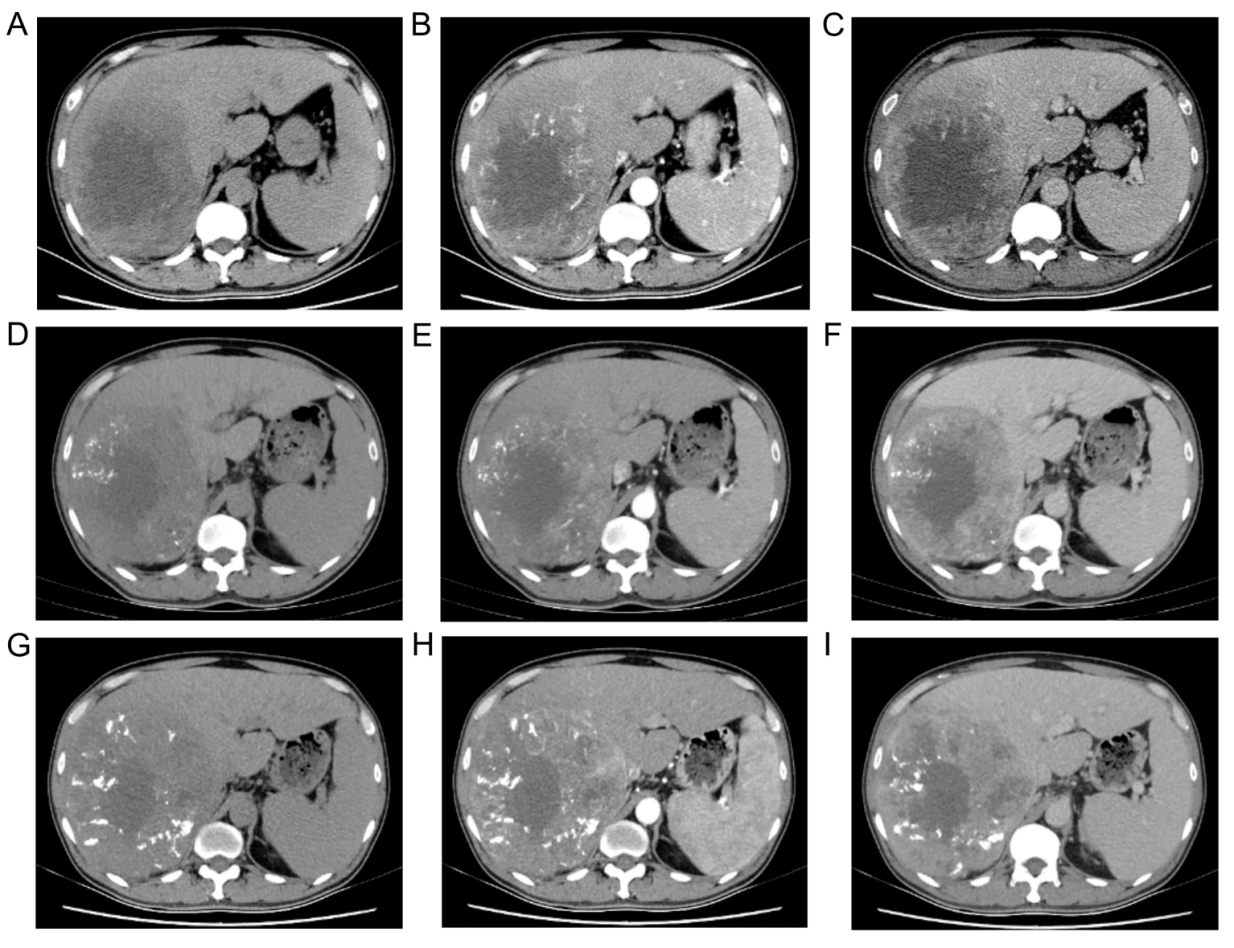
A 42-year-old male with alcohol-associated cirrhosis history. The pre- transarterial chemoembolization (TACE) dynamic CT shows a 130-mm tumor in the right hepatic lobe with obvious increase in arterial phase and decreased in portal phase (**A**-**C**). The dynamic enhanced CT after the initial TACE (**D**-**F**) and the second TACE (**G**-**I**) show a viable tumor >50%.

**Figure 3:**
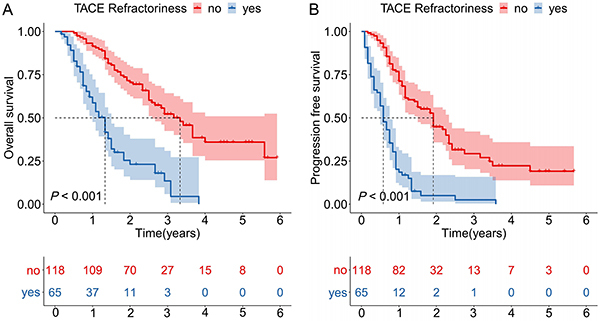
Overall survival (**A**) and progression-free survival (**B**) curves of transarterial chemoembolization (TACE) refractory patients and TACE non-refractory patients in the training cohort.

Early TACE refractoriness patterns are summarized in Table 3. In the training cohort, 35.5% of patients developed early TACE refractoriness. No significant difference was found in the proportion of patients with early TACE refractoriness between the training cohort and the validation cohorts (internal cohort, 36.3%, P=1.000; external cohort, 37.0%, P=0.966). A representative case illustrating early TACE refractoriness is shown in Figure 2 (consecutive viable lesion >50%). The Kaplan-Meier curves were built to explore the influence of early TACE refractoriness on the prognosis of patients in the training cohort (Figure 3). Significant associations were observed between early TACE refractoriness and worse OS (P*<*0.001) as well as PFS (P*<*0.001). The median OS of all patients, TACE refractory patients, and TACE non-refractory patients was 38 months (95%CI: 24.0-36.0), 16 months (95%CI: 13.1-18.9), and 40 months (95%CI: 33.3-46.7), respectively. The median PFS of all patients, TACE refractory patients, and TACE non-refractory patients was 14 months (95%CI: 11.6-16.4), 7 months (95%CI: 5.2-8.8), and 23 months (95%CI: 19.1-26.9), respectively.

**Table 3:** The patterns of early transarterial chemoembolization (TACE) refractoriness.

Characteristics	Training cohort(n=183)	Internal validation cohort (n=80)	External validation cohort (n=54)
TACE refractoriness, n (%)	65 (35.5)	29 (36.3)	20 (37.0)
Viable lesions >50%, n (%)	39 (21.3)	18 (22.5)	12 (22.2)
Vascular invasion, n (%)	10 (5.5)	5 (6.3)	4 (7.4)
Extrahepatic spread, n (%)	10 (5.5)	4 (5.0)	3 (5.6)
Presence of new lesions, n (%)	8 (4.4)	2 (2.5)	1 (1.9)
Elevation of AFP, n (%)	31 (16.9)	11 (13.8)	13 (24.1)

AFP: alpha fetoprotein.

### Inflammatory markers cut-off values

**Figure 4:**
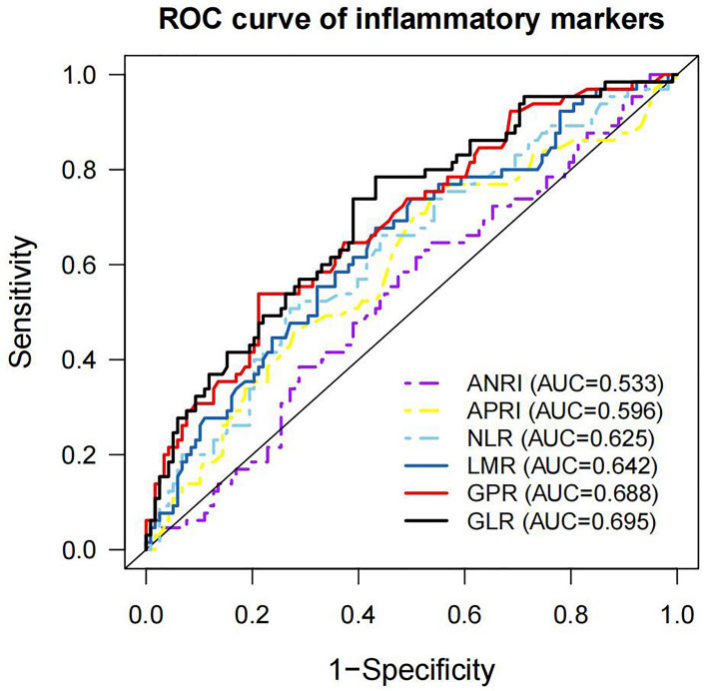
ROC curves of inflammatory markers in training cohort. ANRI: aspartate aminotransferase-to-neutrophil ratio index; APRI: aspartate aminotransferase-to-platelet ratio index; NLR: neutrophil-to-lymphocyte ratio; LMR: lymphocyte-to-monocyte ratio: GPR: gamma-glutamyl transpeptidase-to-platelet ratio; GLR: gamma-glutamyl transpeptidase-to-lymphocyte ratio.

In the training cohort, the mean NLR was 3.30±5.20, the mean GLR was 135.12±173.79, the mean GPR was 1.05±1.16, the mean LMR was 3.05±1.70, the mean APRI was 0.39±0.36, and the mean ANRI was 17.32±15.64 (Table 1). The optimal cut-off values of NLR (2.85; AUC=0.625), GLR (70.85; AUC=0.695), GPR (1.00; AUC=0.688), LMR (2.91; AUC=0.642), APRI (0.22; AUC=0.596), and ANRI (11.35; AUC=0.533) were calculated by the ROC curves according to the best Youden index (Figure 4). The population was divided into two subgroups based on the cutoffs.

### Independent predictors for early TACE refractoriness

In the univariate analysis, variables with a significance level below 0.05 and a variance inflation factor lower than 5 including tumor size, tumor number, bilobular invasion, BCLC stage, AFP, monocyte, neutrophil, AST, GGT, NLR, GLR, GPR, LMR, APRI, and ANRI were selected for further multivariate analysis. The outcomes of multivariate analysis demonstrated that tumor size (P*<*0.001; OR=3.893, 95%CI: 1.947-7.785), bilobular invasion (P=0.007; OR, 4.207, 95%CI: 1.492-11.867), AFP (P=0.035; OR=2.757, 95%CI: 1.075-7.070), and APRI (P=0.007; OR=4.422, 95%CI: 1.504-13.000) were independent predictors for early TACE refractoriness (Table 4).

**Table 4:** Univariate and multivariate analysis for independent risk indicators of early transarterial chemoembolization (TACE) refractoriness in the training cohort.

Characteristics	TACE non-refractory group (n=168)	TACE refractory group(n=95)	P value	
			Univariate	Multivariate
Age	63.2±12.1	61.1±13.4	0.279	-
Gender (%)			0.186	-
Male	96 (81.4)	58 (89.2)		
Female	22 (18.6)	7 (10.8)		
Tumor size (%)			<0.001	<0.001 (OR=3.893; 95%CI: 1.947-7.785)
≤50 mm	67 (56.8)	12 (18.5)		
50-100 mm	41 (34.7)	25 (38.5)		
>100 mm	10 (8.5)	28 (43.0)		
Tumor number (%)			<0.001	-
Solitary	64 (54.2)	20 (30.8)		
2-3	38 (32.2)	17 (26.2)		
>3	16 (13.6)	28 (43.0)		
Bilobular invasion (%)			<0.001	0.007 (OR=4.207; 95%CI: 1.492-11.867)
No	101 (85.6)	36 (55.4)		
Yes	17 (14.4)	29 (44.6)		
BCLC stage (%)			<0.001	-
0	21 (17.8)	1 (1.5)		
A	57 (48.3)	23 (35.4)		
B	40 (33.9)	41 (63.1)		
Ascites (%)			0.620	-
Absence	91 (77.1)	48 (73.8)		
Presence	27 (22.9)	17 (26.2)		
Liver cirrhosis (%)			0.700	-
Absence	42 (35.6)	25 (38.5)		
Presence	76 (64.4)	40 (61.5)		
AFP (%)			0.002	0.035 (OR=2.757; 95%CI: 1.075-7.070)
≤400 ng/mL	100 (84.7)	42 (64.6)		
>400 ng/mL	18 (15.3)	23 (35.4)		
Child Pugh score (%)			0.638	-
A	96 (81.4)	51 (78.5)		
B	22 (18.6)	14 (21.5)		
Hepatitis B (%)			0.286	-
Absence	66 (55.9)	31 (47.7)		
Presence	52 (44.1)	34 (52.3)		
Monocyte (10ˆ9/L)	0.492±0.243	0.720±0.786	0.002	-
Lymphocyte (10ˆ9/L)	1.40±0.538	1.41±0.634	0.931	-
Neutrophil (10ˆ9/L)	3.51±2.20	4.23±1.89	0.034	-
Platelet (10ˆ9/L)	166±83.0	193±111	0.069	-
ALT (U/L)	39.2±39.9	46.1±34.4	0.244	-
AST (U/L)	45.6±36.6	64.8±50.7	0.009	-
GGT (U/L)	117±124	249±296	<0.001	-
NLR (%)			0.002	-
<2.85	86 (72.9)	32 (49.2)		
≥2.85	32 (27.1)	33 (50.8)		
GLR (%)			<0.001	-
<70.85	67 (56.8)	14 (21.5)		
≥70.85	51 (43.2)	51 (78.5)		
GPR (%)			<0.001	-
<1.00	93 (78.8)	30 (46.2)		
≥1.00	25 (21.2)	35 (53.8)		
LMR (%)			0.002	-
>2.91	67 (56.8)	21 (32.3)		
2.91	51 (43.2)	44 (67.7)		
APRI (%)			0.006	0.007 (OR=4.422; 95%CI: 1.504-13.000)
<0.22	54 (45.8)	16 (24.6)		
≥0.22	64 (54.2)	49 (75.4)		
ANRI (%)			0.143	-
<11.35	55 (45.8)	23 (35.4)		
≥11.35	63 (53.4)	42 (64.6)		

Data are reported as mean and SD or number and percent. Fisher’s test or *t*-test. AFP: alpha fetoprotein; ALT: alanine aminotransferase; AST: aspartate aminotransferase; ANRI: aspartate aminotransferase-to-neutrophil ratio index; APRI: aspartate aminotransferase-to-platelet ratio index; NLR: neutrophil-to-lymphocyte ratio; LMR: lymphocyte-to-monocyte ratio: GGT: γ-glutamyl transpeptidase; GPR: gamma-glutamyl transpeptidase-to-platelet ratio; GLR: gamma-glutamyl transpeptidase-to-lymphocyte ratio.

### Development and validation of early TACE refractoriness prediction model

**Figure 5:**
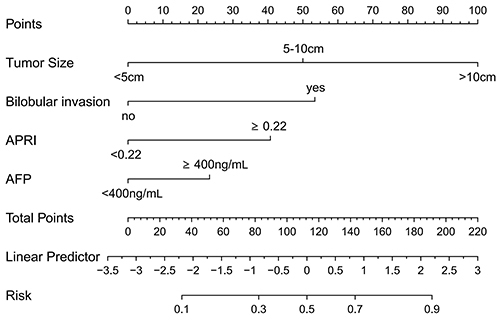
Diagnostic nomogram for predicting early transarterial chemoembolization (TACE) refractoriness. The values on the risk-axis were matched to the total points, which is the sum of points of four variables, reflecting the predicted incidence of developing early TACE refractoriness. APRI: aspartate aminotransferase-to-platelet ratio index; AFP: alpha-fetoprotein.

**Figure 6:**
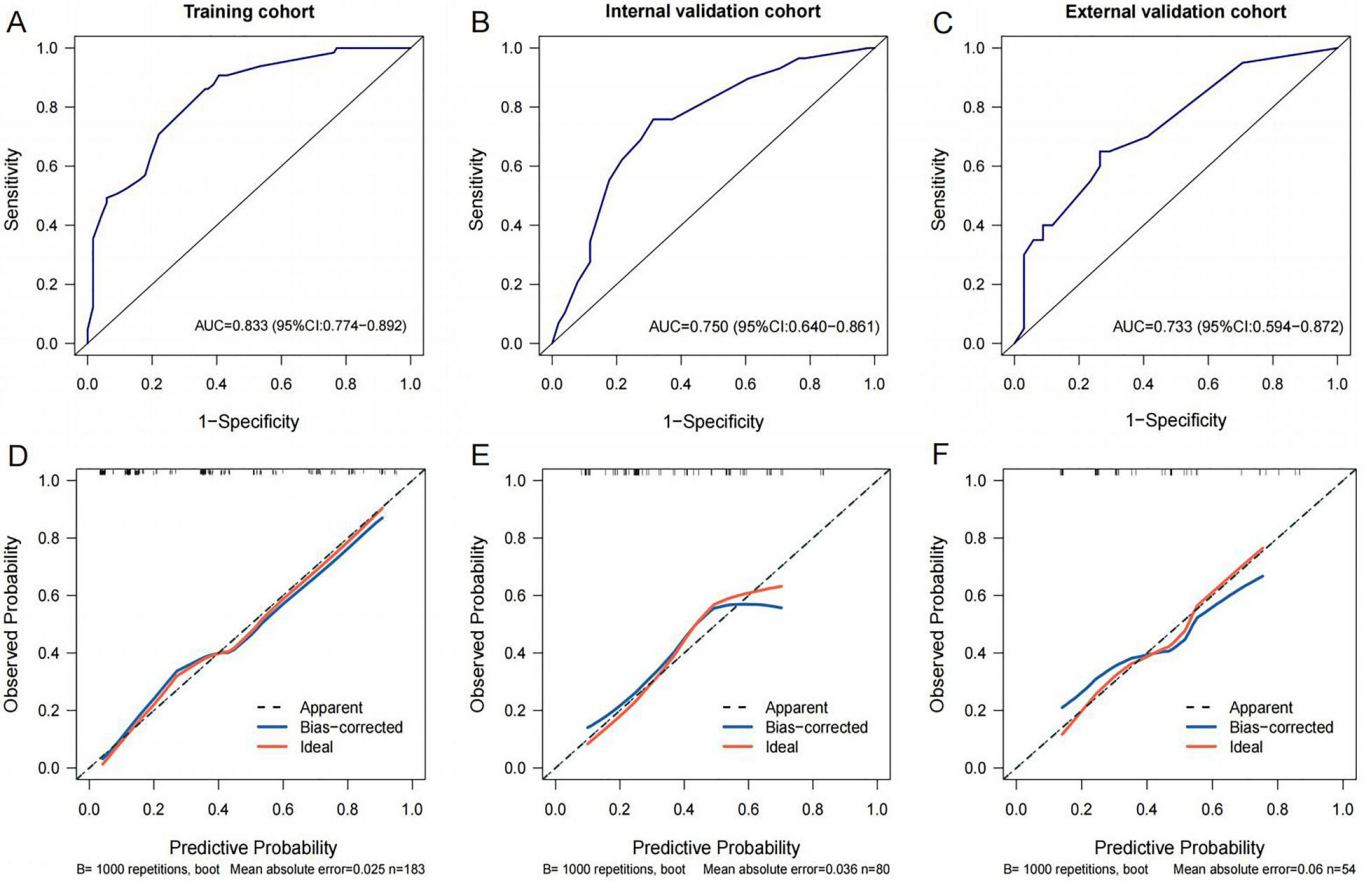
Receiver-operating characteristic (ROC) curves of early transarterial chemoembolization (TACE) refractoriness diagnostic model in the training cohort (**A**), internal validation cohort (**B**), and external validation cohort (**C**). Calibration curves of early TACE refractoriness diagnostic model in the training cohort (**D**), internal validation cohort (**E**), and external validation cohort (**F**).

The diagnostic model and nomogram were constructed to estimate the incidence of early TACE refractoriness based on tumor size, bilobular invasion, AFP, and APRI (Figure 5). The model demonstrated good performance in all three cohorts (Figure 6A-C). The area under the curve (AUC) for risk of early TACE refractoriness was 0.833 (95%CI: 0.774-0.892; sensitivity [TPR]=0.908, specificity [TNR]=0.593, accuracy [ACC]=0.705) in the training cohort and 0.750 (95%CI: 0.640-0.861; TPR=0.759, TNR=0.686, ACC=0.713) in the internal validation cohort and 0.733 (95%CI: 0.594-0.872; TPR=0.650, TNR=0.735, ACC=0.704) in the external validation cohort. Furthermore, subgroup analysis showed that our model had good predictive ability in HCC patients with different tumor stages and liver function status (Supplementary Figure S1). The calibration curves demonstrated a strong agreement between the predicted values of the model and actual values of the model (Figure 6D-F). The Hosmer-Lemeshow test results revealed no statistical significance (training cohort: P=0.825; internal validation cohort: P=0.687; external validation cohort: P=0.901), indicating that the model provided a favorable fit to the data.

## Discussion

Cancer-related chronic inflammation is a common characteristic of cancer (12). Inflammatory ratios are biomarkers of the relationship between the tumor stromal microenvironment and the immune response after TACE treatment, and they have the potential to identify early TACE refractoriness (13). A few of recent retrospective studies have evaluated the correlation between NLR and TACE refractoriness (14,15). Nevertheless, those studies included only one inflammatory marker and thus may not represent the comprehensive value of inflammatory markers in predicting TACE refractoriness. In this paper, we studied six common markers, including NLR, GLR, GPR, LMR, APRI, and ANRI and observed statistically significant differences in multiple inflammatory markers with univariate analysis. However, with multivariate analysis, only APRI, tumor size, bilobular invasion, and AFP were identified as independent risk indicators for early TACE refractoriness.

Intrahepatic tumor control and liver reserve have been demonstrated as the most vital prognostic factors in patients with HCC (16). Adequate preservation of liver function is considered as important as achieving objective treatment response (17). Several studies have shown that ineffective repeat TACE may lead to liver function deterioration, missed opportunities for systemic treatments, and decreased survival time (7,18,19). So far, researchers have established a variety of TACE refractoriness predictive models. Hu et al. (15) first established a pre-TACE model, containing vascularization pattern, major tumor size, AFP, GGT, and ALBI grade to identify patients who had a high risk of experiencing refractoriness. Patients were assigned to six subgroups with different estimated possibilities of TACE refractoriness according to risk scores (range from 0 to 19.5). Similarly, Chen et al. (20) built a TACE refractoriness score with tumor number and bilobular invasion, with scores >3.5 points indicating a higher likelihood of TACE refractoriness. Moreover, some novel models using genomic sequencing data (21,22) and high-dimensional radiomic features (23) have shown promising predictive performance.

Many other plasma markers have also been proven to be related to TACE refractoriness. Hiraoka et al. (24) reported that patients with more than two positive tumor markers, containing AFP (≥100 ng/mL), fucosylated alpha fetoprotein (≥10%), and des-gamma-carboxy prothrombin (≥100 mAU/mL) may have a higher probability to develop TACE refractoriness. Furthermore, research showed that the serum level of arginase-1, an enzyme in the urea cycle, in TACE refractory patients was significantly lower than that in non-refractory patients (36.55 *vs* 54.22 ng/mL, P*<*0.05) (25). In addition, Kim et al. (26) found that high expression levels of plasma circulating microRNA (miR)-122 (>100) are associated with early TACE refractoriness. In another similar study, they demonstrated that the combination of plasma miRNA-21 (≥2.5), miRNA-26a (≥1.5), and miRNA-29a-3p (<0.4) expression was found to be an independent predictor of early TACE refractoriness (P=0.031) (27). However, most of these markers have not gained popularity in the preoperative routine blood tests.

Inflammatory markers, such as blood routine tests and biochemical indexes, have the advantages of being inexpensive and easily obtained at outpatient clinics. Our study evaluated the diagnostic efficacy of serum inflammatory markers in identifying early TACE refractory patients and developed a TACE refractoriness model based on tumor size, bilobular invasion, APRI, and AFP level, which demonstrated a considerable connection with early TACE refractoriness in our intricate multivariate analysis. Specifically, size and number are both major determinants of tumor burden and are related to poor overall survival in HCC patients who underwent TACE (28). In addition, a previous study has proven that tumor size and tumor number are useful predictive indicators for complete response and recurrence after TACE treatment (29). However, in our study, tumor number was not identified as an independent predictor for early TACE refractoriness. The possible reason was that some patients with multiple lesions were classified as TACE non-refractory group since a good treatment response was observed in the target lesions after two sessions of TACE. Bilobular HCC is aggressive, and curative resection is often not feasible. HCC patients with bilobular invasion are more likely to develop extrahepatic metastasis (30). AFP is the most commonly referenced biomarker for auxiliary diagnosis of HCC (31). Moreover, high levels of AFP have been associated with portal vein tumor thrombosis (32), early recurrence (33), and adverse prognosis (34) in patients with HCC.

To our knowledge, this was the first research to consider APRI as a predictive indicator for early TACE refractoriness in patients with early/intermediate stages HCC. APRI, which is calculated with serum AST and platelet levels, is an effective tool for estimating the severity of cirrhosis and has been shown to be comparable to liver biopsy (35). A meta-analysis of 28 cohort studies demonstrated that high APRI levels were associated with poorer OS and disease-free survival in patients with HCC (11). High APRI indicates increased AST and/or decreased platelet count. The serum concentration of AST, mainly present in mitochondria of hepatocytes, increases when the liver is severely damaged. The deterioration of liver function caused by TACE-related toxicity (36) is one possible reason for TACE refractoriness. Platelet count serves as a marker of liver cirrhosis severity. Advanced cirrhosis with portal hypertension causes hypersplenism, which results in platelet consumption (37). Low platelet count is correlated with more severe liver disease, poor survival, and increased perioperative mortality in resectable HCC (38,39). In addition, low platelet count has been found to be a significant factor in predicting postoperative recurrence and intrahepatic distant recurrence (40). To sum up, inflammatory ratios are biomarkers of the correlation between the hepatic tumor therapeutic response and the inflammatory response. Our research preliminarily demonstrated the potential value of APRI in predicting early TACE refractoriness.

However, the present study was not without its constraints. Firstly, the retrospective nature of the study design potentially led to selection bias. Secondly, the lack of a clearly defined optimal cut-off value for APRI resulted in a wide range of reported cutoffs. Efforts should therefore be made to standardize the definition of increased APRI in larger multicenter cohorts. Thirdly, although an independent cohort was collected for external validation, the comparatively small sample size may introduce statistical errors. Finally, as our study specifically focused on patients without venous tumor thrombus or distant metastasis, the TACE refractoriness model might not be applicable to HCC patients in BCLC stage C or D.

### Conclusion

In conclusion, our findings suggested that TACE refractory patients had significantly shorter OS and PFS compared to TACE non-refractory patients. Tumor size, bilobular invasion, APRI, and AFP level were recognized as independent risk factors for early TACE refractoriness. Furthermore, we have developed and validated a novel TACE refractoriness model that can help identify early TACE refractoriness and guide therapeutic strategies for HCC patients in BCLC stages 0, A, and B.

## Supplementary Material

Click here to view [pdf].
