# Supplementary material for: Diagnostic value of serum inflammatory markers in predicting early refractoriness of transarterial chemoembolization in patients with Barcelona Clinic Liver Cancer Stage 0, A, and B hepatocellular carcinoma
Source: Braz J Med Biol Res. 2024 Sep 6;57:e13661. doi: 10.1590/1414-431X2024e13661 (PMC11379351; doi:10.1590/1414-431X2024e13661)

**Figure S1.** Subgroup analysis of model prediction performance in BCLC 0&A groups (**A**), BCLC B group (**B**), Child Pugh A group (**C**), and Child Pugh B group (**D**).

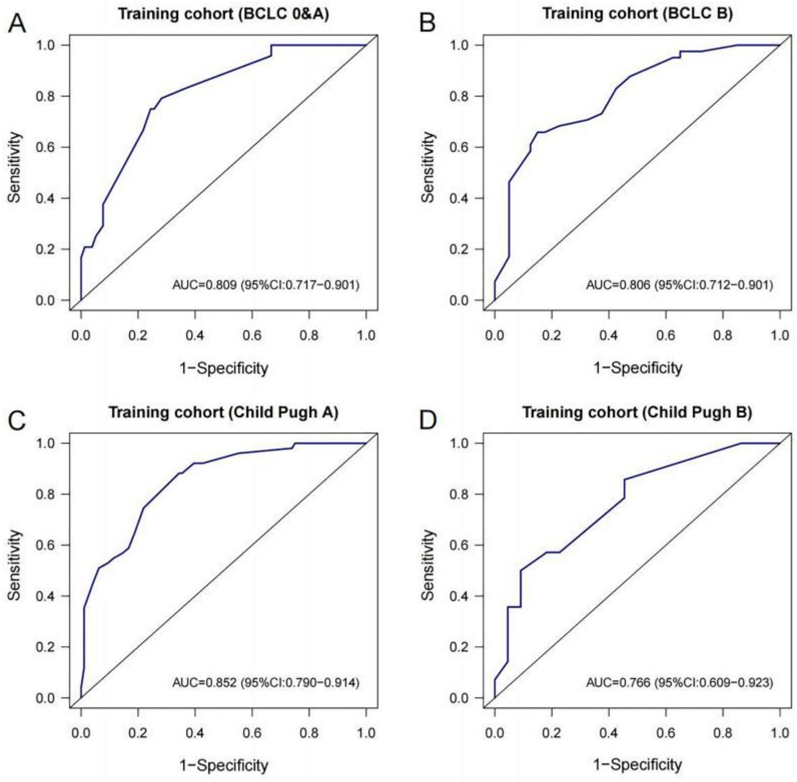

Supplement: Supplementary file 1 [file 1414-431X-bjmbr-57-e13661-suppl.pdf]
